# Assessing the Quality of Life of Oral Submucous Fibrosis Patients: A Cross-Sectional Study Using the WHOQOL-BREF Tool

**DOI:** 10.3390/ijerph18189498

**Published:** 2021-09-09

**Authors:** Abdul Bari Memon, Aneela Atta Ur Rahman, Kashif Ali Channar, Muhammad Sohail Zafar, Naresh Kumar

**Affiliations:** 1 Medical Research Centre, Liaquat University of Medical and Health Sciences, Jamshoro 76090, Pakistan; drabmemon@yahoo.com (A.B.M.); draarahman@hotmail.com (A.A.U.R.); 2 Faculty of Community Medicine and Public Health Sciences, Shaheed Mohtarama Benazir Bhutto Medical University, Larkana 77150, Pakistan; 3 Department of Oral and Maxillofacial Surgery, Institute of Dentistry, Liaquat University of Medical and Health Sciences, Jamshoro 76090, Pakistan; kashifomfs@gmail.com; 4 Department of Restorative Dentistry, College of Dentistry, Taibah University, Al Madinah, Al Munawwarah 41311, Saudi Arabia; drsohail_78@hotmail.com; 5 Department of Dental Materials, Islamic International Dental College, Riphah International University, Islamabad 44000, Pakistan; 6 Ishrat Ul Ebad Khan Institute of Oral Health Sciences, Dow University of Health Sciences, Karachi 74200, Pakistan

**Keywords:** oral submucous fibrosis, overall health, quality of life, reliability

## Abstract

The aim of the present study was to evaluate the quality of life (QoL) of oral submucous fibrosis (OSMF) patients using the World Health Organization Quality of Life-BREF (WHOQOL-BREF) questionnaire. This cross-sectional study was conducted at the Department of Oral and Maxillofacial Surgery, Liaquat University of Medical and Health Sciences (LUMHS), Jamshoro. We used the consecutive sampling technique to recruit patients who were clinically diagnosed with OSMF (*n* = 112). Data were collected using the WHOQOL-BREF questionnaire, which contains a total of 26 questions. The first two questions, related to overall QoL and overall health status, were evaluated separately. The remaining questions (3–26), which represented four domains—physical, psychological, social, and environmental health—were evaluated separately. Patients were asked questions in their native language (Urdu). The relationship between these four domains of life was evaluated with gender, age categories, functional staging, and habit duration using the independent *t*-test to determine statistical significance. Cronbach’s Alpha was used to assess the reliability of the WHOQOL-BREF domains. The overall QoL of the OSMF patients was considerably poor, and the majority of the patients were unsatisfied with their oral health status. The age variable significantly affected the scores of all domains except for social relationships, whereas habit duration and functional staging of OSMF did not significantly affect the scores of all domains. The domains of the WHOQOL-BREF questionnaire (translated into the Urdu language) showed good reliability, except for social relationships.

## 1. Introduction

Oral submucous fibrosis (OSMF) is a precancerous condition. About 7–30% of OSMF patients have been reported with a malignant transformation of this life-threatening condition [1]. The chief presenting complaints of OSMF patients include restricted mouth opening and a burning sensation in the oral mucosa [2,3,4]. Due to this burning sensation, restricted mouth opening, and associated pain, the health of these patients is compromised to a great extent, which may ultimately affect their quality of life (QoL). Moreover, OSMF patients are usually disturbed socially and emotionally due to various dysfunctions, including taste disorders, restricted tongue movements, difficulty swallowing, and speech [5]. There are several predisposing factors associated with OSMF, such as deficiencies of vitamin B, C, and iron; habitual chewing of smokeless tobacco; excessive consumption of spicy foods; genetic mutations; and human papilloma virus (HPV) infection [6,7,8,9,10]. Among these, betel nut chewing is considered the most common risk factor for OSMF [11]. OSMF patients are present around the globe; however, the disease remains remarkably more prevalent in the subcontinent [12,13,14]. The World Health Organization (WHO) reported more than five million OSMF patients worldwide in 2002 [4,15]. Such a large population suffering from OSMF may have a compromised QoL [16]. Moreover, OSMF patients are usually psychologically uncomfortable due to the stress of undergoing malignant transformation [17]. Despite the significant influence of population, increased consumption of tobacco, and greater malignant potential in developing countries, oral health-related quality of life (OHRQoL) is seldom assessed in these patients.

QoL is related to the welfare of humans socially, culturally, and emotionally and is considered an important element of healthcare [18]. The perception of QoL is multicultural, multi-conceptual, and complex; as a result, consensus on its definition is lacking worldwide [19,20]. According to the WHO, QoL has been defined as the “perception of individuals and their status to understand the culture and system in which they live to achieve their goals, expectations, standards and concerns” [21].

QoL is relevant to the care of patients and is a vital health outcome measure. Oral health is a requisite of general health and is an essential element of overall QoL [22]. It is rightly said that if an oral cavity is devoid of any problems with performing functions in daily life, then individuals can achieve their social roles without any physical, mental, or social obstacles [23]. If there is any ailment in the oral cavity resulting in pain, discomfort, or functional difficulties while eating or speaking, it may jeopardize the patients’ self-confidence and social communication [24].

It has been observed that a healthy population enjoys a more satisfactory QoL than an affected one. In recent years, public health researchers have devoted more attention to QoL. More interest has been shown by investigators in assessing the QoL of the general population because it has become a major outcome measure in health-related research across the world [18,25,26].

There are a variety of tools available for assessing OHRQoL: Oral Impacts on Daily Performances (OIDP), the Oral Health Impact Profile (OHIP), the University of Washington Quality of Life Questionnaire (UWQOL), the Chronic Oral Mucosal Disease Questionnaire (COMDQ), the World Health Organization Quality of Life-BRE (WHOQOL-BREF), and Oral Health Related Quality of Life-UK (OHRQoL-UK) [12]. These measuring tools can be disease-specific or generic to measure specific oral health conditions and overall oral health, respectively [5,27]. The WHOQOL-BREF questionnaire is considered a reliable, valid, and multi-cultural measuring tool of QoL, and it covers four domains of life: physical, psychological, and environmental health, and social relationships. Moreover, a couple of other items associated with overall QoL and general health status are also included in this tool [28,29]. This questionnaire has been translated and validated into over 40 languages around the world—including Urdu, the national language of Pakistan, which was adopted in the current study for data collection [30,31].

Numerous studies have been conducted in both general and diseased populations, including OSMF patients [14,18,25,26,32,33,34,35,36]. The studies conducted in OSMF patients have focused on its etiology, pathology, genetic mutation, malignant potential, and various available treatment modalities, but its impact on different domains of patients’ lives has been investigated in a limited way or has not been investigated properly. OSMF is a chronic disorder affecting the oral cavity, the first part of the digestive system; therefore, it is necessary to evaluate its consequences on QoL. In the literature, a few research groups have evaluated the reliability and validity of the WHOQOL-BREF questionnaire in the Urdu language [31,37]. However, to the best of our knowledge, none of the research groups have assessed the OHRQoL of OSMF patients using the WHOQOL-BREF questionnaire; this warrants a comprehensive assessment of its psychometric properties. Therefore, the aim of the present study was to assess the QoL of OSMF patients. For this purpose, we used the WHOQOL-BREF questionnaire in the native language of the residents. The hypothesis of the current study is that if QoL is severely affected by OSMF, then patients with OSMF would have lower scores on the WHOQOL-BREF.

## 2. Materials and Methods

### 2.1. Patient Inclusion and Exclusion Criteria

This cross-sectional study was conducted at the Department of Oral and Maxillofacial Surgery, LUMHS Jamshoro, Pakistan, after approval from the institutional research ethics committee. Ethical permission was sought from the Research Ethics Committee of the University before the commencement of this study (Reference: NO.LUMHS/REC/-640; 26/12/17).

The patients were told about the purpose and protocol of the study before their recruitment, and a written consent form was then obtained in the local language from the participants before the commencement of study. The OSMF patients were diagnosed clinically. All patients fulfilling the inclusion criteria were recruited using a consecutive sampling technique.

The inclusion criteria for the selection of study participants were as follows: OSMF patients having mouth opening from 15 mm to 35 mm, age equal to or above 18 years, both male and female, and patients willing to give up habits of chewing tobacco.

Patients with the following criteria were excluded: displaying other causes of limited mouth opening (temporo-mandibular problems or pericoronitis, trauma, previously surgically treated patients), patients with any other mucosal disease (leukoplakia, erythroplakia, oral squamous cell carcinoma), patients with major systemic medical problems, and those with psychiatric illness.

### 2.2. Data Collection Procedure

Two clinicians individually evaluated the patients to confirm the diagnosis of OSMF. The patients were enrolled after consensus was achieved by both of the clinicians, who were experts in the field of oral and maxillofacial surgery and had more than five years’ clinical experience. Demographic information was recorded on the designed proforma. A detailed history of the disease was recorded. A thorough clinical examination was performed on all recruited participants. Measurements of mouth opening (inter-incisal mouth opening) were performed following the guidelines described by Dijkstra et al. [38] Briefly, the patients were asked to open their mouths as wide as possible without feeling any discomfort or pain while keeping their heads in an upright resting position. The mouth opening was measured (in mm) from the incisal edge of the upper central incisor to the incisal edge of the lower central incisor using a digital vernier caliper (Kawasaki, Japan). Based on functional staging [39], patients were classified as Stage 1 (>35 mm), Stage 2 (25–35 mm), Stage 3 (15–25 mm), and Stage 4 (<15 mm). The examiner took three measurements, and the mean values were recorded to maintain substantial intra-examiner reliability (kappa values of 0.86).

The WHOQOL-BREF questionnaire is comprised of 26 questions. The first two questions are related to the assessment of overall QoL and overall health status, whereas the remaining questions, from 3–26, represent four domains: “Physical Health = Domain 1,” “Psychological Health = Domain 2,” “Social Relationships = Domain 3,” and “Environment = Domain 4” [40,41]. Each patient first had the questions clearly explained to them using their native language. Based on the events of the previous two weeks, the patients were asked to respond to questions for a particular score. The answers for each item were recorded on a Likert-type scale signifying scores from 1–5, where 1 and 5 denoted the minimum and maximum effects, respectively. A higher total of points scored signifies a higher QoL in the relevant domain.

### 2.3. Statistical Analysis

Statistical Package for Social Sciences (SPSS) version 16 (IBM Corp., Armonk, NY, USA) was used for the analysis of data sets. Qualitative variables, such as age group, gender, duration of chewing habits, and stages of OSMF, were calculated as frequency and percentage. Quantitative variables, such as QoL score, satisfaction with health, and domains of life, were presented as mean and standard deviation. Gender, age categories, functional staging, and habit duration were analyzed with physical, psychological, social, and environmental domains of life using an independent *t*-test to highlight their statistical significance. The reliability of the WHOQOL-BREF domains were assessed using Cronbach”s Alpha. The significance of each variable was highlighted when its *p*-value was revealed as <0.05.

## 3. Results

The QoL variations in all four domains for both age groups (median 40) are shown in Table 1. QoL was statistically better in those over 40 years old in all domains (*p* < 0.05) except “social health” (*p* = 0.591). Females showed a good QoL in “physical,” “psychological,” and “environmental health” when compared to males, but in the “social relationship” domain, females showed a poor quality of life compared to their male counterparts (*p* = 0.001). The habit duration and functional stage of OSMF did not exhibit any significant differences within each domain of life (Table 1).

**Figure 1:**
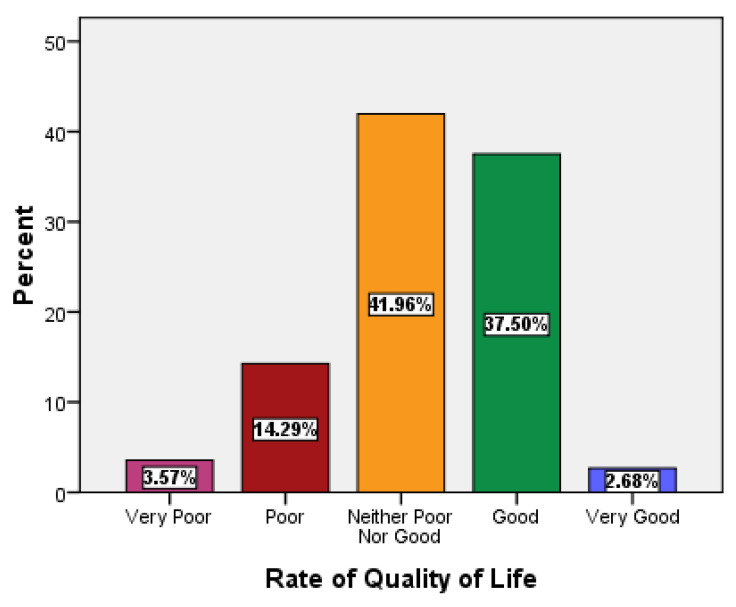
Descriptive statistics or characteristics of overall rate of QoL.

When asked the first question, “How would you rate your quality of life,” the responses were 37.50% for “good,” 42% for “neither poor nor good,” and 14% for “poor” (Figure 1).

**Figure 2:**
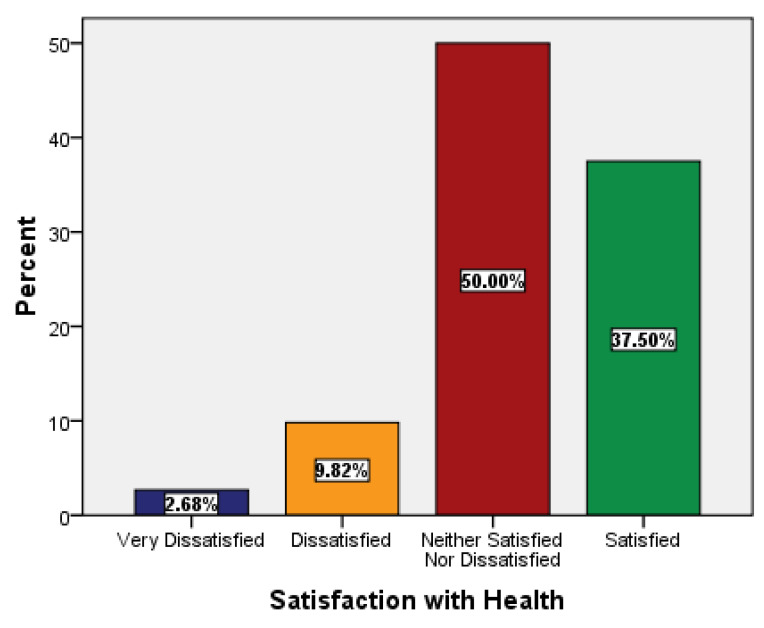
Descriptive statistics of overall satisfaction with health.

The responses for the 2nd question related to satisfaction with health were 50% for “neither satisfied nor dissatisfied” and 37.50% for “satisfied” (Figure 2).

Cronbach’s α coefficient determined the level of internal consistency as 0.880 for the items of the Urdu WHOQOL-BREF tool used in our study. While looking into patients’ self-assessment data, the mean scores of “overall self-reported QoL” and “self-rated satisfaction with current health” were found to be 3.21 ± 0.85. and 3.22 ± 0.73, respectively. With regard to QoL domains, the highest mean score was observed in the environmental domain at 27.09 ± 4.32, followed by the physical health domain at 25.96 ± 4.46, the psychological domain at 20.62 ± 2.81, and finally, the social relationship domain, with the least score, at 10.75 ± 1.49 (Table 2).

The Urdu version of the WHOQOL-BREF exhibited an average item score in the range of 2.60 to 4.02 (Table 2). The domains of the WHOQOL-BREF scale highlighted the internal reliability coefficients above 0.70, apart from the “social relationships” domain of life (0.628), as shown in Table 3.

## 4. Discussion

In this study, the WHOQOL-BREF tool was employed for the first time in Pakistan to assess the QOL of OSMF patients. The WHOQOL-BREF tool was preferred to other similar tools including OHIP-14, the 16-item UK Oral Health-related Quality of Life measure (OHQOLUK-16), the Oral Health Impact Profile-16 (OHIP-16), and OHRQoLUK, [12,40,41] as these tools only focus oral health statuses. In addition, the University of Washington Quality of Life Questionnaire (UW-QoLv4) and Postoperative Symptom Severity questionnaire (PoSSe) were also not selected, as the present study employed no intervention for the comparison of QoL before and after treatment [42]. The psychometric properties of this tool have been widely used in the general population [43,44] as well as in various clinical populations [45,46], excluding OSMF patients. In this study, the WHOQOL-BREF, a generic type of questionnaire, was preferred over a disease-specific questionnaire, as it is a reliable tool that has been translated into many languages and is good for a comparison of QoL in both normal and diseased populations [5,27,28,29,30,31,35,36]. Ailments from oral cavities are not lethal but can affect eating and speaking functions and contribute to one’s QoL [47]. The disease, which could disturb the routine of daily life, may have an adverse effect on general well-being.

OHRQoL is a newly emerging field of research in the last few decades, so it has an important role in clinical practice and dental research. Furthermore, it is firmly believed that OSMF is a chronic disease that affects oral health due to fibrosis of oral mucosa and limited mouth opening (LMO). According to the published literature, the impact of OSMF on the OHRQoL of many patients is evident [17,48,49,50]. This area demands more study, particularly in South Asian countries, due to the greater prevalence of habit-related OSMF.

In this study, out of 112 OSMF patients, 61 were under the age of 40. This trend is a serious matter because the prevalence of OSMF is increasing among young individuals, which is alarming due to its potential association with developing oral cancer in the younger population. This is also highlighted in a review of OSMF in a pediatric population [51], which emphasized it as a central, important public health issue to prevent this pre-malignant lesion. In the present study, QoL worsened significantly with aging in all domains except social health. In a previous study by Chaudhry et al. [36], QoL worsened significantly in all domains. The reason for such trends may be attributed to various factors, including increased responsibilities, age-related diseases, stresses related to family care, financial issues, anxiety, family, or employment matters, and psychological fears.

As far as gender is concerned, most authors have observed a greater victimization of males in OSMF [52,53,54]; this is also the case in this study. Our study found a male predilection with am M/F ratio of 1.7:1. The study depicted a slightly better QoL among females in the physical, psychological, and environmental domains than males. However, in the social relationship domain, males have a slightly better QOL. Notably, it was statistically significant in the social relationship domain. Meanwhile, Sahmadhavi N et al. [55] have observed using OHIP as a tool of measurement that both genders are equally affected, whereas Caglayan et al. [56] observed more female predilection in oral diseases in general. This study points out that social relationships are statistically better in males. Such gender-based differences may be attributed to social and cultural values, as females are usually shyer to explain their desire for sex and are culturally bound to avoid personal relationships [57,58].

The chewing of smokeless products is one of the main causative factors of OSMF; studies have shown that the chewing of areca nut in the form of quid is the root cause [59,60,61]. The findings of this study have shown that there was no significant effect of the habit duration of chewing products on QoL. This is in contradiction with the results of Chauhdary et al. [36], who reported a significant worsening of QoL in patients who consumed chewing tobacco for a longer duration. The difference in QoL might be due to variations in manufacturing products, genetic predilection, nutritional deficiency, and oral hygiene habits.

All QOL domains were statistically insignificant in relation to functional stages (mouth opening). This is not in agreement with the results of Chaudhry et al. [35], who observed that all the domains of QOL were significantly affected with progression of the disease from mild to moderate to severe results. The difference could be due to not including all the functional stages of mouth opening. These findings suggest that until mouth opening is severely affected, there is no significant effect on QoL. Thus, such patients do not quit their habits and visit doctors unless the patients are severely affected by LMO or fear of the development of oral cancer and thus cannot enjoy life in a better way. In the present study, when asked to rate QoL and satisfaction with health, 37.50% responded “good” and 37.50% responded “satisfied,” which is not in agreement with the results of a study conducted in India, where 83% responded as “good” and 68% responded as being “satisfied” [35]. This difference could be due to variations in sample size, area, culture, health facilities, and financial issues. Our findings indicate lower scores on the WHOQOL-BREF tool, while the overall QoL of the patients was considerably poor, and the same patients were not satisfied with their health. Thus, the hypothesis was accepted. These findings are in agreement with studies conducted by Chaudhry et al. [35] and Tadakamadla et al. [62] in India, by Suliman et al. [34] in Sudan among diseased patients, and by Lodhi FS et al. [63] in Pakistan among the general population. This might be due to the lack of facilities in terms of better living conditions, access to hospitals, transport, quality education, security, physical mobility, entertainment, and shopping centers.

In the current study, the highest mean scores (27.09 ± 4.32) were observed for the environmental domain and the lowest scores (10.75 ± 1.49) for the social relationship domain. This is in contradiction with the study results of Chaudhry K et al. [35], who used the same tool to assess the impact of OSMF on QoL, where they observed the highest score (15.65 ± 2.23) in the social domain and the lowest score (14.33 ± 2.06) in the social domain. The reasons for these differences could be the variations in the study samples analyzed, cultural changes, social differences, financial issues, and the recruitment procedure, in which all the participants of OSMF were recruited with restrictions on clinical severity based on mouth opening. The lowest mean scores for the social domain could be attributed to the lesser social mingling of patients due to tobacco chewing, difficulty in maintaining friendly relationships owing to aesthetic issues, and less support from the family in our recruited patients. The highest average QoL scores of the environmental domain highlight the average financial resources to meet their needs, namely, proper health and social care: suitable home environment, accessibility, and quality, participation in and opportunities for recreation/leisure activities, proper opportunities for acquiring new information and skills, satisfaction from the available transport and physical environment (pollution/noise/traffic/climate), and good physical safety and security.

The internal reliability for the items of the Urdu WHOQOL-BREF was revealed as 0.880, which indicates a good reliability. Some other studies also showed similar results for QoL, including assessment of QoL in Greek populations [64], Spanish populations [65], Koreans with physical impairment [66], and the healthy and diseased population of Iran [31]. Apart from the “social relationships” domain, internal reliability coefficients for all other domains of the WHOQOL-BREF scale were above 0.70. This agrees with an international study that revealed the internal consistency for the “social relationships” domain to be 0.62 in total [43]. The subscale “social relationships” comprises only three items: sexual activity, personal relationships, and social support. This subscale exhibited low internal reliability, which could be due to the relatively fewer number of items [67]. Moreover, a low alpha value is likely due to cultural and personal life questions in our population. In addition, financial issues could have prevented OSMF patients from participating in normal social activities. Furthermore, the communication of females in our society is relatively low.

Furthermore, the corrected item-total correlation was identified in a range between 0.11 and 0.95, which is far greater than the recommended value of 0.20 [68], except for the negative feeling item of the psychological health domain. This is because the majority of OSMF patients have a blue mood or become depressed, despairing, and nervous after the confirmed diagnosis of the disease, as the likelihood of malignant transformation is high. Oral health awareness programs should be arranged for different communities, especially in areas where OSMF is endemic and consumption of smokeless tobacco products is at a higher level, because chewing tobacco is the main culprit behind the development of pre-malignant and malignant oral diseases. Public awareness may be increased through mass media and social media for tobacco cessation and in the depiction of the hazards of harmful products. Psychological counseling may be required to quit such products to improve QoL.

Only two functional stages were incorporated in this study, which is one of the limitations of this study. Further studies with a large sample size and inclusion of all stages may corroborate the results. Another limitation of the study is the lack of awareness of the target audience about the OSMF-specific questionnaire. The strength of the study is that the translated questionnaire has already been validated in the native language, i.e., Urdu, for the assessment of QoL in the Pakistani population. Self-reported data are always considered a limitation, and there may be recall bias to fill in the answers, keeping in mind just the previous two weeks. There was no sufficient sample size or single-centered hospital-based study, so the results may not be generalizable to the whole population of OSMF. Therefore, multi-centered research should be undertaken with the involvement of a larger sample size using more clinical parameters. We would like to suggest that awareness programs should be designed for OSMF patients, and screening should also be done to diagnose cases at early stages so that precautionary measures may be taken and QoL may be improved for many. It appears that future research work will focus on the development of disease-specific questionnaires for OSMF rather than relying on the generic QoL tools.

## 5. Conclusions

The overall QoL of the OSMF patients was considerably poor, and the majority of the patients were unsatisfied with their oral health status. The age variable significantly affected the scores of all domains except social relationships, whereas habit duration and functional staging of OSMF did not significantly affect the scores of all domains. The domains of the WHOQOL-BREF questionnaire (translated into the Urdu language) showed good reliability, except for the social relationships domain of life.

## Figures and Tables

**Table 1:** Difference in mean scores of age groups, gender, habit duration, and functional stages with four domains of life by Student’s *t* test.

	Physical Health	Psychological Health	Social Relationships	Environmental Health
	Mean ± SD	Mean ± SD	Mean ± SD	Mean ± SD
AGE GROUPS				
Less than <40 Years (*n* = 61)	26.78± 4.46	21.24 ± 2.63	10.81 ± 1.62	28.00 ± 4.39
More than >40 Years (*n* = 51)	24.89 ± 4.31	19.88 ± 2.87	10.66 ± 1.32	26.01 ± 4.01
*p*-value	0.032	0.010	0.591	0.015
GENDER				
Male (*n* = 74)	25.56 ± 4.72	20.37 ± 2.92	11.09 ± 1.50	26.54 ± 4.31
Female (*n* = 38)	26.73 ± 3.86	21.10 ± 2.57	10.07 ± 1.23	28.18 ± 4.17
*p*-value	0.191	0.198	0.001	0.056
HABIT DURATION				
<Less than 5 years (*n* = 41)	25.70 ± 4.49	20.87 ± 2.67	10.60 ± 1.49	27.25 ± 4.61
>More than 5 Years (*n* = 71)	26.00 ± 4.41	20.42 ± 2.87	10.80 ± 1.47	26.94 ± 4.16
*p*-value	0.733	0.416	0.492	0.721
FUNCTIONAL STAGES				
M-2 Mild (*n* = 32)	25.34 ± 4.94	20.59 ± 2.55	10.53 ± 1.75	26.65 ± 5.02
M-3 Moderate (*n* = 80)	26.21 ± 4.27	20.63 ± 2.93	10.83 ± 1.37	27.27 ± 4.02
*p*-value	0.355	0.941	0.329	0.496

SD = Standard Deviation.

**Table 2:** Reliability of WHOQOL-BREF domain scores and two stand-alone questions.

Domains of Life	Minimum	Maximum	Mean	Std. Deviation
DOM-1	16.00	34.00	25.96	4.46
DOM-2	16.00	25.00	20.62	2.81
DOM-3	7.00	14.00	10.75	1.49
DOM-4	18.00	35.00	27.09	4.32
Rate of Quality of Life	1	5	3.21	0.85
Satisfaction with Health	1	4	3.22	0.73
Cronbach’s α coefficient	0.880			

DOM = Domain.

**Table 3:** Internal consistency of WHOQOL-BREF domains in patients with OSMF.

WHOQOL BREF	Mean	Standard Deviation	Corrected Item Total Correlation	Cronbach’s Coefficient	Cronbach‘s Alpha if Item Deleted
Domain 1				0.805	
Physical pain	2.60	1.16	0.487		0.806
Medical treatment	2.66	1.03	0.423		0.810
Enough energy	3.40	0.67	0.702		0.756
Get around	3.38	0.78	0.566		0.774
Sleep satisfaction	3.68	0.50	0.393		0.803
Daily living activities	3.39	0.67	0.767		0.746
Capacity of work	3.48	0.68	0.681		0.759
Domain 2				0.776	
Life enjoyment	3.53	0.69	0.662		0.706
Meaningful life	3.65	0.65	0.665		0.708
Able to concentrate	3.50	0.63	0.703		0.700
Bodily appearance	3.49	0.50	0.531		0.747
Satisfaction with him/herself	3.35	0.59	0.765		0.689
Negative feelings	3.11	0.94	0.119		0.881
Domain 3				0.628	
Personal relationship	3.40	0.52	0.623		0.335
Sex life	4.02	0.52	0.362		0.630
Support from friends	3.33	0.86	0.436		0.636
Domain 4				0.952	
Feeling of safety	3.46	0.68	0.917		0.939
Physical environment	3.35	0.56	0.719		0.952
Money for needs	3.47	0.78	0.901		0.942
Information in daily life	3.42	0.61	0.868		0.943
Leisure activities	3.23	0.50	0.499		0.963
Satisfaction with living place	3.44	0.61	0.828		0.945
Satisfaction health services	3.35	0.59	0.955		0.937
Satisfaction with transport	3.38	0.60	0.899		0.941

## Data Availability

The data presented in this study are available on the request from the corresponding author.

## Abbreviations

QoL	Quality of Life
OSMF	Oral Submucous Fibrosis
WHOQOL-BREF	World Health Organization Quality of Life-BREF
LUMHS	Liaquat University of Medical and Health Sciences
HPV	Human Papilloma Virus
WHO	World Health Organization
OIDP	Oral Impacts on Daily Performances
OHIP	Oral Health Impact Profile
UWQOL	University of Washington Quality of Life Questionnaire
COMDQ	Chronic Oral Mucosal Disease Questionnaire
OHQoL-UK	Oral Health Related Quality of Life-UK
OHRQoL	Oral Health Related Quality of Life
SPSS	Statistical Package for Social Sciences
SD	Standard Deviation
DOM	Domain
LMO	Limited Mouth Opening
